# A Built-In Mechanism to Mitigate the Spread of Insect-Resistance and Herbicide-Tolerance Transgenes into Weedy Rice Populations

**DOI:** 10.1371/journal.pone.0031625

**Published:** 2012-02-16

**Authors:** Chengyi Liu, Jingjing Li, Jianhua Gao, Zhicheng Shen, Bao-Rong Lu, Chaoyang Lin

**Affiliations:** 1 State Key Laboratory of Rice Biology, Institute of Insect Sciences, College of Agriculture and Biotechnology, Zhejiang University, Hangzhou, China; 2 Ministry of Education Key Laboratory for Biodiversity and Ecological Engineering, Institute of Biodiversity Science, Fudan University, Shanghai, China; University of Leeds, United Kingdom

## Abstract

**Background:**

The major challenge of cultivating genetically modified (GM) rice (*Oryza sativa*) at the commercial scale is to prevent the spread of transgenes from GM cultivated rice to its coexisting weedy rice (*O. sativa* f. *spontanea*). The strategic development of GM rice with a built-in control mechanism can mitigate transgene spread in weedy rice populations.

**Methodology/Principal Findings:**

An RNAi cassette suppressing the expression of the bentazon detoxifying enzyme CYP81A6 was constructed into the T-DNA which contained two tightly linked transgenes expressing the Bt insecticidal protein Cry1Ab and the glyphosate tolerant 5-enolpyruvylshikimate-3-phosphate synthase (EPSPS), respectively. GM rice plants developed from this T-DNA were resistant to lepidopteran pests and tolerant to glyphosate, but sensitive to bentazon. The application of bentazon of 2000 mg/L at the rate of 40 mL/m^2^, which is approximately the recommended dose for the field application to control common rice weeds, killed all F_2_ plants containing the transgenes generated from the Crop-weed hybrids between a GM rice line (CGH-13) and two weedy rice strains (PI-63 and PI-1401).

**Conclusions/Significance:**

Weedy rice plants containing transgenes from GM rice through gene flow can be selectively killed by the spray of bentazon when a non-GM rice variety is cultivated alternately in a few-year interval. The built-in control mechanism in combination of cropping management is likely to mitigate the spread of transgenes into weedy rice populations.

## Introduction

Genetically modified (GM) crop with insect-resistant and herbicide-tolerant traits have been extensively cultivated in many parts of the world, providing greater opportunities to meet the challenges of the global food security [1]. As an important world crop providing stable food and nutrition for nearly one half of the global population, rice (*Oryza sativa*) has been a primary target for genetic engineering As a consequence, many GM rice lines with different traits (e.g., insect resistance and herbicide tolerance) have been developed [2], [3], [4]. Now, some of the GM rice lines are in the pipelines waiting for commercialization, which is particularly manifested by the issuance of biosafety certificates to two insect-resistant *Bt* GM rice lines by Chinese authority. However, environmental biosafety is a concern for the extensive cultivation of GM crops. One major biosafety concern over the commercial cultivation of GM rice is the spread of transgenes from GM rice to weedy and wild rice through transgene flow and the subsequent introgression [5], [6]. The spread of insect-resistance and herbicide-tolerance transgenes into weedy and wild rice populations may create serious weed problems [6].

To prevent and significantly reduce the opportunities of transgene flow from GM rice to populations of wild rice relatives (*Oryza* spp.), spatial isolation between GM rice and its wild relatives is strongly proposed [7]. However, such an isolation strategy is not applicable to weedy rice (*O. sativa* f. *spontanea*) as it usually occurs simultaneously with cultivated rice in the same fields. Thus, the prevention of transgene flow from rice cultivars to weedy rice plants is nearly impossible via any physical isolation. Novel strategic designs are necessary to mitigate transgene introgression and spread into weedy rice populations. Bentazon is a selective herbicide which has been used for effective weed control in rice fields for over a decade. Rice is naturally resistant to bentazon due to its expression of the detoxification enzyme CYP81A6 [8]. Previously, our laboratory reported a novel method to create GM rice with a built-in mechanism for controlling the spread of transgenes [9]. In that strategic design, the transgenes of interest were tagged with an RNAi cassette suppressing the expression of CYP81A6. Any GM rice line developed with this method will be sensitive to bentazon, and thus can be selectively killed by the spray of bentazon.

Weedy rice is one of the most noxious weeds in rice fields all over the world [10], [11]. This weed can cause great yield losses and quality reduction of rice due to its seed shattering and contamination of rice grains during harvest. It is labour cost to manually control weedy rice and nearly impossible to kill weedy rice by chemical means because weedy rice belongs to the same species of cultivated rice. In this study, we developed an insect-resistant and glyphosate-herbicide tolerant GM rice line that is sensitive to bentazon. This was achieved by constructing a T-DNA with two tightly linked transgenes expressing the Bt insecticidal protein Cry1Ab, the glyphosate tolerant 5-enolpyruvylshikimate-3-phosphate synthase (EPSPS), and an RNAi cassette suppressing the expression of the bentazon detoxifying enzyme. We demonstrated in the field experiment that the crop-weed rice hybrid lineages with the transgenes could be killed selectively by the spray of bentazon. Based on this experiment, we consider that any hybrid progeny resulted from transgene flow from the GM rice to weedy rice could be selectively killed by the use of bentazon under the regular weed control in rice fields when a non-GM rice variety is cultivated after cultivation of the GM rice for a few years. Such a strategic design of a transgenic construct coped with a particular cropping and weed management can mitigate the rapid spread of transgenes in weedy rice populations and ensure the long-term and safe cultivation of the GM rice.

## Results

### Development of transgenic rice lines

**Figure 1:**
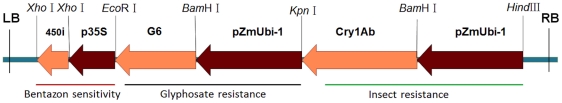
Diagram of the T-DNA fragment of the plasmid p1300-450i-G6-Cry1Ab. pZmUbi-1, *Zea mays* polyubiquitin-1 promoter; *Cry1Ab*, insecticidal crystal protein Cry1Ab; *G6*, the 5-enolpyruvylshikimate-3-phosphate synthase isolated from *Pseudomonas putida* fused with chloroplast transit peptide at the N-terminus (gb:EU169459); p35S, cauliflower mosaic virus 35S promoter; 450i, the inverted repeat sequence of the 207 bp fragment of *CYP81A6*; LB and RB, left and right border of the T-DNA.

A binary T-DNA transformation plasmid p1300-450i-G6-Cry1Ab [Fig. 1], containing a *Cry1Ab* expression cassette for insect resistance, an *EPSPS* expression cassette for glyphosate tolerance, and RNAi cassette suppressing the expression of *CYP81A6* which detoxifies herbicide bentazon, was transformed into a local rice variety “Xiushui 110” (*O. sativa* subsp. *japonica*) by the *Agrobacterium*-mediated method. About 40 independent transgenic lines were obtained from the transformation. All these transgenic lines survived on the rooting media containing 0.1 mM glyphosate.

### Analysis of transgenic lines

**Figure 2:**
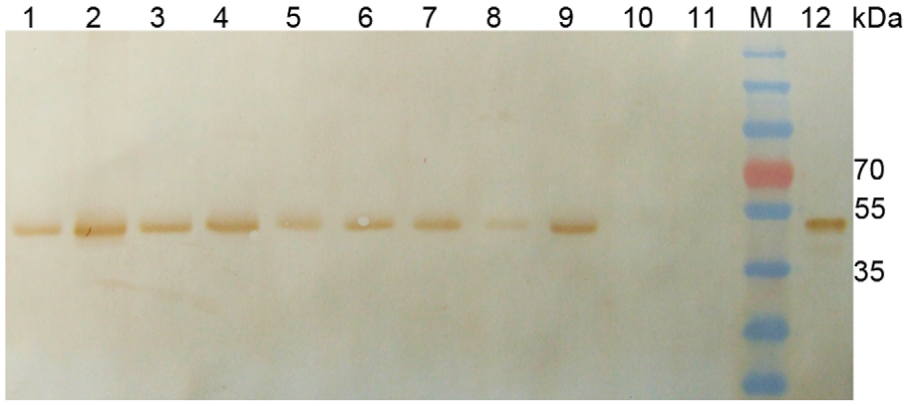
Western analysis of G6 (EPSPS) expression in transgenic T0 plants. Lane 1 to 9, different lines of transgenic rice; Lane10 and 11, non-transformed rice as the negative control; Lane12, positive control.

The transgenic rice lines were assayed for their glyphosate tolerance, bentazon sensitivity, and insect resistance. To test the glyphosate tolerance, the T_0_ plants of the transgenic lines were cultured in a greenhouse and sprayed with 20 mM glyphosate. All the transgenic lines survived from the herbicide spray while all the non-transgenic controls were killed by the spray. This was expected as these transgenic lines survived from the rooting media containing glyphosate. To examine the expression of the glyphosate tolerant *EPSPS* gene *G6* (gb: EU169459), transgenic lines were examined by immunoblotting with a polyclonal antibody specific to the EPSPS. An EPSPS protein with an estimated molecular mass of 42 kDa was detected in all the transgenic lines but not the non-transgenic control plants (Fig. 2).

To determine insecticidal activity of the transgenic rice lines, leaves of the transformed lines were subjected to assay with the 1^st^ instar larvae of rice leaf-folders (*Cnaphalocrosis medinalis* Guenee). Of the 40 transgenic lines, 21 were found with insignificant damage to their leaves, whereas leaves of all the non-transgenic rice lines were seriously damaged by the larvae. This result was expected as the transgenic rice expressing Cry1 has been reported previously to be resistant to rice leaf folder [12], [13]. The transgenic lines with low or no resistance to the leaf-folder larvae were likely due to the low expression of the insecticidal protein.

**Figure 3:**
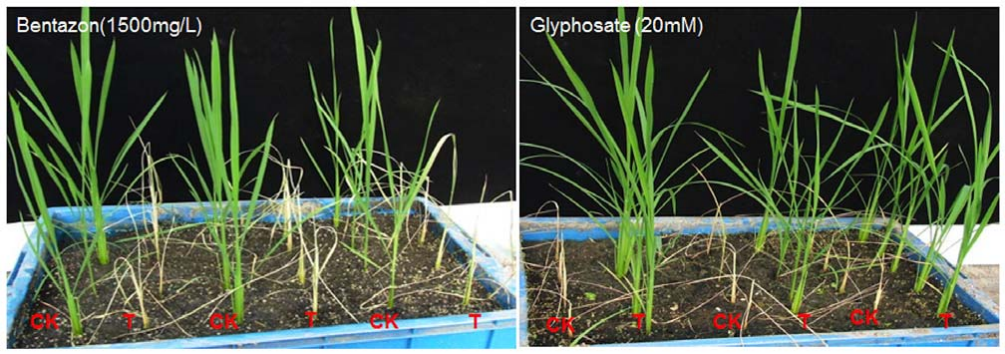
Sensitivity test of transgenic rice plants to bentazon or glyphosate. The T0 transgenic rice plants (T) along with non-transgenic (CK) ones were cultured in greenhouse and sprayed with 1500 mg/L bentazon (A) or 20 mM glyphosate (B). The pictures were taken 7 days after the spray.

**Figure 4:**
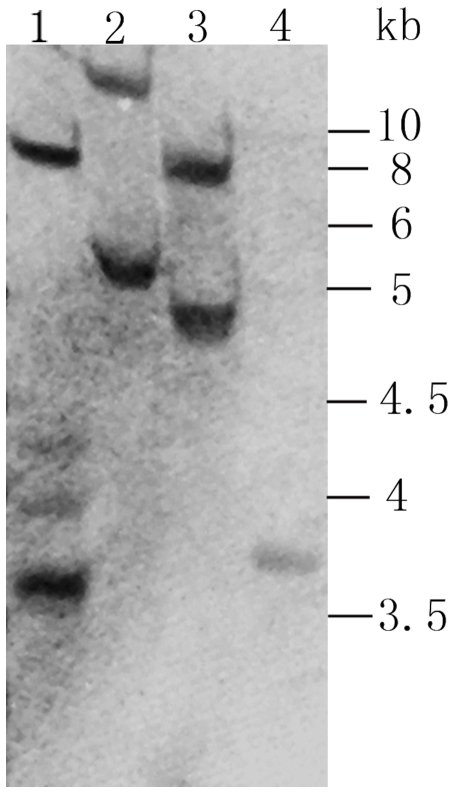
Southern analysis of transgenic rice. The genomic DNA was isolated from transgenic rice plants and them hybrid with a probe prepared with G6 (EPSPS). The restriction enzyme used for genomic DNA digestion was *Kpn*I. Lanes 1–4, event CGH-2, CGH-5, CGH-9 and CGH-13.

The 21 insect resistant rice lines were further tested for their sensitivity to bentazon. Parts of the T_0_ plants from each of the insect resistant lines were sprayed with bentazon at 1500 mg/L in the greenhouse. Ten transgenic lines were killed by bentazon spray at the 7^th^ days, suggesting that these 10 events were highly sensitive to bentazon (Fig. 3). To search for a transgenic line with only a single copy of the transgene, four of these 10 sensitive lines were selected for Southern analysis. Among the 4 lines analyzed, the CGH-13 line appeared to have only a single copy of the transgene insertion (Fig. 4). The high expressers of the insecticidal protein appeared to be more likely to have multiple copies of the transgenes. Line CGH-13 was used for the study of hybridization (gene flow) with weedy rice.

### Development of crop-weedy rice hybrids with transgenes

Seeds derived from self-pollinated T_0_ plants (CGH-13) were collected and germinated on a field seed bed. The T_1_ plants were expected to have segregation for their transgenes because the T_0_ plants were not homozygous. To determine the plants without transgenes, glyphosate was sprayed on the T_1_ plants. About 25% of the T_1_ plants were killed by the spray of glyphosate. The remaining T_1_ plants resistant to glyphosate were then transplanted into a greenhouse and the pollens from these plants were collected to pollinate the weedy rice strains (PI-63 and PI-1401).

**Figure 5:**
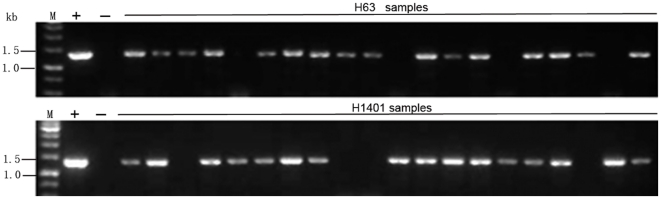
PCR detection of transgene in F2 hybrid plants of cultivated rice and weedy rice. M, 1 kb ladder of DNA markers; +, positive control; -, negative control; H63 samples, F2 hybrids of CGH13×PI-63; H1401 samples, F2 hybrids of CGH13×PI-1401.

Approximately 80 F_1_ plants of CGH-13×PI-63 and CGH-13×PI-1401 were obtained from the crosses. These crop-weedy rice hybrid plants were sprayed with glyphosate to kill those without transgenes at the age of about 30 days after seed germination. The glyphosate resistant F_1_ hybrid plants containing the transgene were self-pollinated and the seeds were harvested to generate F_2_ plants. To determine the existence of the transgenes in F_2_ populations, we planted the F_2_ population and randomly selected sufficient number of plants for analysis using PCR to determine the existence of the transgenes. The ratio of transgene-positive versus -negative plants was about 3∶1 (Fig. 5). This ratio was expected for F_2_ populations derived from self-pollination of the heterozygous transgenic F_1_ plants.

### Reaction of cultivated and weedy parents, GM rice line, and F_2_ segregated populations to herbicides

**Figure 6:**
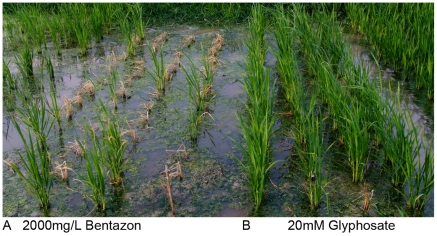
Field trial for F2 hybrid population. The F2 hybrid plants of CGH13×PI-63 were planted in field and sprayed with 2000 mg/L of bentazon(A) or 20 mM of glyphosate(B) at 40 ml/m^2^. This picture was taken 10 days after spraying.

The seedlings of the cultivated (Xiushui-110) and weedy parents (PI-63 and PI-1401), GM line (CGH-13), and populations of F_2_ hybrids were transplanted individually into the field to test their sensitivity to bentazon or glyphosate. Twenty days after transplanting, the plants were sprayed with either 2000 mg/L bentazon or 20 mM glyphosate, respectively. The plants were checked eight days after the spray. All the cultivated rice and weedy rice parents were sensitive to glyphosate but resistant to bentazon. However, the majority of the F_2_ plants were sensitive to bentazon but resistant to glyphosate (Fig. 6). PCR detection was conducted to analyze the F_2_ plants which survived from the herbicide spray. No transgene was detected in 30 bentazon tolerant F_2_ plants, indicating that both homozygous and heterozygous transgenic plants were sensitive to bentazon and were killed. All the F_2_ plants containing the transgenes survived from the spray of glyphosate.

Results from the herbicide spray experiment showed that all the transgene-positive plants in the F_2_ populations were tolerant to glyphosate but sensitive to bentazon (Table 1). In contrast, the cultivated parents, weedy parents, and the transgene-negative plants of the F_2_ populations were sensitive to glyphosate but tolerant to bentazon spray (Table 1).

**Table 1:** Reactions to glyphosate or bentazon of the cultivated and weedy rice parents, GM rice line, and F_2_ segregate populations with or without transgenes.

Plant material	Code	No. of plants examined	Tolerant to glyphosate**	Tolerant to bentazon**
Parental rice variety	Xiushui-110	200	−	+
Weedy rice-1	PI-63	200	−	+
Weedy rice-2	PI-1401	200	−	+
GM rice line	CGH-13	120	+	−
F_2_ hybrid lineage-1	CGH-13/PI-63			
TP-pop*		94	+	−
TN-pop*		30	−	+
F_2_ hybrid lineage-2	CGH-13/PI-1401			
TP-pop*		92	+	−
TN-pop*		30	−	+

*TP-pop, transgene-positive F_2_ segregate population; TN-pop, transgene-negative F_2_ segregate population.

**+, tolerant to glyphosate or bentazon; − sensitive to glyphosate or bentazon.

## Discussion

Our experiments demonstrated that the insect-resistant and herbicide-tolerant GM rice line with the built-in selective mechanism is sensitive to bentazon and could be killed by the spray of bentazon at the regular dosage. In contrast, the parental non-GM rice variety was not affected by the spray of bentazon. Similarly, the GM crop-weedy rice hybrid lineages containing the transgenes are also sensitive to bentazon and can be selectively terminated. The feature of this built-in control mechanism of the GM rice will be very useful in preventing the introgression and spread of the transgenes into weedy rice populations.

Weedy rice usually coexists with cultivated rice and is highly competitive in rice fields [14]. Weedy rice is widely distributed in the rice-planting regions all over the world, particularly in East, South and Southeast Asia, South and North America, and Southern Europe [10], [15]. In China, weedy rice is now commonly found in rice fields of many provinces [16]. Weedy rice was effectively managed and became negligible 15–20 years ago because of the extensive manual transplanting and intensive human labour for weed control in general [17]. However, with the adoption of direct seeding and/or other no-till technologies, in addition to less inputs of weed control labour, weedy rice has reoccurred as a major weedy problem in Jiangsu, Hainan provinces and northeast parts of China [18], [19], [20], [21]. This weed has already caused a major damage to rice production by reducing rice grain yield and quality in Northeastern China [22]. Therefore, the effective control of weedy rice populations becomes very important for the sustainable production of rice crop in China, as well as in other rice planting regions worldwide.

Herbicide-based weed management is generally the most popular method for weed control in the direct-seeded rice fields. However, it is very difficult to control weedy rice by the use of selective herbicides because weedy rice is essentially the same biological species as cultivated rice. Most herbicides currently available in the markets are not functioning selectively against weedy rice and leave cultivated rice not being affected [23]. Therefore, the development of herbicide tolerant GM rice provides an ideal solution. In fact, glufosinate-resistant and glyphosate-resistant rice lines have been developed and are ready to enter commercial markets [2]. However, the spread of the herbicide tolerant transgenes into weedy rice will limit the commercial application of the GM rice because the coexisting weedy rice cannot be selectively killed by these herbicides. Thus, a built-in mechanism of selective termination is highly desirable to control the spread of transgenes into weedy rice.

Weedy rice and cultivated rice are the same species, and they are sexually highly compatible with nearly no reproductive barriers [24]. Therefore, transgenes conferring tolerance to an herbicide from cultivated rice will inevitably move to weedy rice populations occurring in the same fields. Once the herbicide resistant GM rice is commercially released for cultivation, the transgene will surely move into the coexisting weedy rice populations through recurrent transgene flow and the subsequent introgression [6]. Herbicide-resistant weedy rice plants that have acquired transgenes can be rapidly accumulated when the same type of herbicide is repeatedly applied for weed control. As a consequence the control of weedy rice by herbicide spray will become extremely difficult. A previous report based on modeling studies already predicted that herbicide resistance genes could become common in weedy rice populations within 3–8 years of continuous rice cropping [25].

Our transgenic strategy with the built-in control mechanism [9] can easily mitigate the accumulation and spread of transgenes in weedy rice populations when combined with the appropriate cropping management. Results from this study demonstrate that the insect-resistant and herbicide-tolerant GM rice with the built-in control mechanism and its resulted crop-weedy rice hybrids can be selectively killed by the spray of bentazon at 2000 mg/L. This is the recommended dose by the manufacturer that can effectively control weeds in rice field but safe for cultivated non-GM rice. The maximum dose of bentazon that regular rice can tolerate is about 5800 mg/L [8]. The ideal scenario is that glyphosate can be regularly used to control weeds (including weedy rice) in rice fields when GM rice containing such herbicide tolerant transgenes is cultivated. If transgenes are accumulated in weedy rice populations to a certain threshold level through recurrent gene flow, which cannot be avoided, and cause considerable damage to rice production due to the increased tolerance of weedy rice plants to glyphosate, non-GM rice varieties can be cultivated in combination with the application of bentazon to control rice weeds, particularly weedy rice containing transgenes.

By the application of such a built-in mechanism combined with cropping management through alternating cultivation of GM and non-GM rice varieties according to the necessity, weedy rice with or without transgenes will be effectively removed or at least managed to a minimal level by spraying bentazon or glyphosate alternately. In addition, the alternating cultivation between GM and non-GM rice varieties can slow down the development of resistance to transgenes that exposed to continuous high selection pressure. Bentazon can be quickly degraded in soil and is an environment friendly chemical to use [26]. Thus, the spread of transgenes tolerant to herbicides or other biotic stresses in weedy rice can easily be controlled by such a built-in mechanism and appropriate cropping in the agricultural environments. The system can be applied to other crops and their feral species, such as oil-seed rape versus weedy *Brassica* species and sugar beet versus weedy beets.

## Materials and Methods

### T-DNA plasmid construction

The cassette for RNAi suppression of *CYP81A6* was described previously [9]. Briefly, the 207 bp fragment of the 5′ end of *CYP81A6* was obtained by PCR from rice genomic DNA using the primer 450F (5′CTCGAGCAGTGCACCAGAGTCACAGAAACACATCACAC (an *Xho*I site was attached and underlined), and 450R (5′AGATCTGCT TCTTGACGAGGTGGAGGTGT, a *Bgl*II site was attached and underlined). A 327 bp fragment of the 5′ end of *CYP81A6* was obtained by PCR from the same rice genomic DNA using the primer 450F, and the primer 450R2 (5′ AGA TCTCGGTGAAGCACTCCCTGGCGCAC, a *Bgl*II site was attached and underlined). This fragment represents 1 to 327 bp of the cDNA from the 5′ end. Both PCR products were cloned into the pMD-T vector (Shanghai Sangon, China), and then released from the T-vectors by digestion with *Xho*I and *Bgl*II. These two fragments were cloned into the T-DNA plasmid vector pCAMBIA1300 which was predigested with *Xho*I and dephosphorylated. The resulted plasmid, which contains a 207 bp inverted repeat sequence of *CYP86A6* for RNA interference, was named as p1300-450i.

The synthetic *EPSPS* gene *G6* conferring glyphosate tolerance was described previously (gb: EU169459). The *Zea mays* polyubiquitin-1 promoter (ZmUbi-1) was obtained by PCR using ZmUbiF-K (5′ TGGGTACCGTGCATGCCTACAGTGCAGCGTGACCCGGTCGTGC, a *Kpn*I site were attached and underlined) and ZmUbiR (5′ GTGGGATCCTCTAGAGTCGACCTGCAGAAGTAACACCAAACAACAG, a *Bam*HI site was attached and underlined). ZmUbi-1 was used for the direct expression of the *G6* gene fused with the chloroplast transit peptide from the acetohydroxyacid synthase of *Z. mays*. The PCR amplified ZmUbi-1 promoter was digested with *Bam*HI and *Kpn*I, the synthetic *G6* gene predigested with *Eco*RI and *Bam*HI, and then cloned into the plasmid p1300-450i predigested with *Eco*RI and *Kpn*I. This binary plasmid was named as p1300-450i-G6.

The rice codon-optimized *Cry1Ab* gene with a terminator was synthesized by Shanghai Sangon Limited Corp. (Shanghai, China), according to the published amino acid sequence (AAQ92302.1) with the following modifications: a *Bam*HI site was introduced at the 5′ end of the *Cry1Ab* gene. A corn phosphoenolpyruvate carboxylase (PEPC) terminator with a *Kpn*I site was added to the 3′ end of the gene. The *Z. mays* polyubiquitin-1 promoter (ZmUbi-1) was used for the direct expression of the *Cry1Ab* gene. This PCR amplified ZmUbi-1 promoter was digested with *Bam*HI and *Hind*III, and then ligated in a three-way ligation to the synthetic *Cry1Ab* gene pre-digested with *Bam*HI and *Kpn*I, and to the plasmid 1300-450i-G6 pre-digested with *Kpn*I and *Hind*III. The resulted binary T-DNA construct was named as p1300-450i-G6-Cry1Ab. Thus, the T-DNA construct includes three cassettes: the *Cry1Ab* expression cassette, the *EPSPS* expression cassette, and the RNA interference cassette (Fig. 1).

### *Agrobacterium*-mediated rice transformation

T-DNA transformation plasmid vector p1300-450i-G6-Cry1Ab was transformed into *Agrobacterium tumefaciens* (LBA4404) by electroporation. A local rice cultivar “Xiushui-110” (*Oryza sativa* L. *ssp. japonica*) was transformed using an *Agrobacterium*-mediated transformation method. Glyphosate (Sigma) of 2–3 mM final concentration was used for selection and regeneration of transgenic calli.

### PCR analysis

PCR method was used to identify the transgenes in the GM rice and the hybrid offsprings between the GM rice and weedy rice. CTAB method [27] was used for the isolation of genomic DNA from rice leaves. The presence of the *G6* gene was determined by amplification of a 1320-bp fragment of the gene using the primer pair G170M-F: 5′-GAGCTCGAGACCAATGGGATCCGGCAGGCTGAACGGCAG-3′ and G170-C: 5′-CTGCTCGAGCTCTCAGCTCTTGCCCTCCTCGG-3′. The PCR products were analyzed by agarose gel electrophoresis.

### Western analysis

Standard western analysis method was carried out to detect the expression of *G6* in transgenic rice plants. Leaf samples collected from transgenic plants as well as non-transgenic control plants were ground in liquid nitrogen and then suspended in SDS sample buffer. After boiled for 10 min, the soluble fractions of these samples were separated by SDS-PAGE and then blotted onto a nitrocellulose membrane. The rabbit antiserum against G6 was used as the first antibody and the alkaline phosphatase-conjugated goat anti-rabbit IgG as the second antibody (Sigma).

### Southern analysis

Genomic DNA was isolated from rice mature leaves using the CTAB method. For Southern blot analysis, 5 µg of genomic DNA was digested with *Kpn*I, which cuts only once at the T-DNA. The digested genomic DNA was size-fractionated by electrophoresis on a 0.7% agarose gel, and then transferred onto a charged nylon membrane (Hybon-N^+^). Hybridization probes specific to *G6* (*EPSPS*) gene was created using the DIG High Prime DNA Labeling and Detection Starter Kit II (Roche) according to the manufacturer's instructions. The DNA of the *G6* probe was amplified by PCR using primers G6-N (5′GGATCCGGGGCCAACGACCTGATCTTCCTG) and G6-C (5′ CTCGAGCTCTTATCAGCTCTTGCCCTCCTCGGCCACCC) from the T-DNA plasmid.

### Spray of herbicides

The target rice plants were sprayed with handheld sprayer at the rate of 40 mL/m^2^ with different concentrations for both bentazon and glyphosate. Bentazon (48% solution) was obtained from Jiangsu Luli Limited (Jiangsu, China). Bentazon was also manufactured and distributed by BASF (Limburgerhof, Germany). For glyphosate tolerance tests, Roundup (41% propylamine salt of glyphosate, Monsanto, USA) was used. Before the herbicides were sprayed on the rice plants, the field was drained to remove the surface water in the field.

### Rice lines, weedy rice strains and crop-weed crosses

A rice variety (Xiushui-110 from Zhejiang Provence of China) and its GM rice line (CGH-13) containing the three transgenes (*EPSPS*, *CYP81A6 interference cassette*, and *Cry1Ab*) were included for herbicide reaction comparison. The two weedy rice strains (PI-63 and PI-1401) obtained from the National Small Grains Collection, USDA-ARS, USA through seed exchange were also used in this study. For the crop-weed hybrid production, the GM rice line CGH-13 used as the pollen donor was crossed with the two weedy rice strains, respectively, between May-October 2009 at the Zhejiang University Farm in Hangzhou, China.
