# Correction: A Built-In Mechanism to Mitigate the Spread of Insect-Resistance and Herbicide-Tolerance Transgenes into Weedy Rice Populations

**DOI:** 10.1371/annotation/f64311a8-b716-4cbb-8b59-32fd1d2188a7

**Published:** 2012-05-15

**Authors:** Chengyi Liu, Jingjing Li, Jianhua Gao, Zhicheng Shen, Bao-Rong Lu, Chaoyang Lin

This correction is issued to acknowledge the previous work by Dr Gressel and his colleagues as we have employed a similar approach to use a second transgene to prevent spread of transgenic traits.

Dr. Gressel and his colleagues proposed the concept of 'transgenic mitigation' (TM) (Gressel, 1999). As part of this approach, mitigator genes are used to decrease fitness in volunteers and hybrid progeny. TM genes such as encoding dwarfing, strong apical dominance to prevent tillering, uniform seed ripening, non-shattering, and/or antisecondary dormancy can be used to reduce the competitive ability of the rare hybrids with weedy or wild rice (Valverde, 2005). Dr Gressel and collaborators also demonstrated that the use of the dwarfing gene in transgenic oilseed rape can effectively mitigate the risk of transgenic spread in field populations when it transfers to non-transgenic varieties (Al-Ahmad, 2006). The built-in control mechanism is similar to the TM strategy. We fused the RNAi cassette to suppress the expression of the bentazon detoxifying enzyme. The results from our study demonstrate that crop-weedy rice hybrids that incorporate a built-in control mechanism can be mitigated by the spray of bentazon.

Gressel J (1999) Tandem constructs; preventing the rise of superweeds. Trends in Biotechnology 17:361-366.

Valverde BE, Gressel J (2005) Implications and containment of gene flow from herbicide-resistant rice (Oryza sativa). 20th Asian-Pacific Weed Science Society Conference. Ho-Chi Minh City Vietnam pp. 63-84.

Al-Ahmad H, Dwyer J, Moloney M, Gressel J (2006) Mitigation of establishment of Brassica napus transgenes in volunteers using a tandem construct containing a selectively unfit gene. Plant Biotechnology Journal 4:7-21. 

